# *Helicobacter pylori* Eradication in Nonulcer Dyspepsia: Does It Really Matter?

**DOI:** 10.4103/1319-3767.39628

**Published:** 2008-04

**Authors:** Ahmad S. AlMalki

**Affiliations:** Division of Gastroenterology, Department of Internal Medicine, Riyadh Military Hospital, Riyadh, Saudi Arabia

**Keywords:** Functional dyspepsia, gastric cancer, *Helicobacter pylori*, nonulcer dyspepsia

## Abstract

One-half of the world's population has *Helicobacter pylori* (*H. pylori*) infection while dyspeptic symptoms affect one-third of the adult population, at least in the Western world. Data from epidemiological studies are controversial in terms of the association of *H. pylori* with non-ulcer dyspepsia (NUD) symptoms. Despite the frequency of occurrence of this clinical condition, no effective therapy exists in treating this disorder. With the strategic aim of treating NUD, a vast amount of evidence has accumulated towards eradicating *H. pylori*, while an equally compelling amount of evidence exists that counters this very strategy. It is, therefore, vital that there is reliable evidence for the efficacy of treatments prescribed to NUD patients. The arguments for and against the eradication of this organism continues unabated. We aim to address both sides of this fundamental divide and present the differing perspective in light of the prevalent evidence.

Nonulcer dyspepsia (NUD) is a common condition and no therapy is dramatically effective in treating this disorder. Rome II definition of dyspepsia ascribes to a chronic or recurrent pain or discomfort centered in the upper abdomen excluding patients with predominant reflux. Functional or nonulcer dyspepsia (NUD) is defined as at least 3 months history of dyspepsia with no obvious structural explanation of symptoms.[1] Dyspepsia is a group of heterogeneous disorders and is further classified into ulcer-like, with symptoms of pain centered in the upper abdomen; dysmotility-like, with symptoms of upper abdominal fullness, early satiety, bloating or nausea; or unspecified, with symptoms not fitting either of these classifications.[2] This particular point makes interpretation of any studies not using this classification difficult.

What complicates the matter further more is the high prevalence rate of associated functional gastrointestinal disorders in this group of patients, like irritable bowel syndrome (IBS) and nonerosive reflux disease. For example, in the US householder study of volunteers where the prevalence of dyspepsia was 13%, one-third of the population had heartburn. However, if heartburn and symptoms of IBS were excluded from the dyspepsia category, only 3% of the population still had a diagnosis of dyspepsia.[3] Undoubtedly, NUD significantly impairs quality of life in affected patients and this was clearly shown in several studies including recently published data from Saudi Arabia.[4]

***Helicobacter pylori*** (***H. pylori***) is a common bacterial pathogen that colonizes the gastric mucosa of over half the world population.[5] ***H. pylori*** is strongly related to peptic ulcer disease; it is found in 80–90% of duodenal ulcer patients, 60–75% of gastric ulcer patients and is also considered a class I gastric carcinogen according to World Health Orgnanization (WHO) classification as well as a recognized cause of gastric MALT lymphomas. However, when it comes to the relation between NUD and ***H. pylori*** the picture is much less clear even in the presence of clear gastritis.

Primary care physicians frequently deal with dyspeptic patients. However, they have minimal or no clear structural explanation when such patients are tested positive for ***H. pylori.*** Eradication of the organism is an all-too-easy approach that many of such physicians and patients alike are willing to adopt. However, a large segment of the physician population seems to be ignorant of the strength of the data supporting this approach in the management of patients with NUD.

This article addresses the issue of treatment of ***H. pylori*** in NUD at several levels: a) the role of ***H.pylori*** in the pathogenesis of NUD; b) the effect of ***H. pylori*** on the symptomatology and outcome of these patients; c) The effect of ***H. pylori*** on the prevention of peptic ulcer disease and more importantly, the prevention of gastric cancer; and d) to highlight the negative aspects of ***H. pylori*** eradication.

## *H. PYLORI* ROLE IN THE EPIDEMIOLOGY AND PATHOGENESIS OF NUD

The relevance of ***H. pylori*** to NUD is uncertain. Initial studies presumed a role of ***H. pylori*** as the organism causes a chronic inflammatory response and has effect on gastric acid secretions,[6] but at the same time other studies did not find any relation between the organism and NUD.[7] In fact, a systematic review of 31 observational studies addressed the epidemiological relevance of ***H. pylori*** to NUD. This review concluded that reports of strong associations in small observational studies without appropriate adjustment for confounding factors were not generally confirmed by large and better-designed studies. Moreover, no studies have been published that can reliably confirm or exclude the existence of any weak associations.[8]

Regarding the pathogenesis of NUD, a number of putative mechanisms have been elucidated, including visceral hypersensitivity, delayed gastric emptying, impaired gastric accommodation, acid sensitivity, and disturbed central perception of peripheral visceral events.[9] It is unclear what role ***H. pylori*** plays in the symptom profile of the NUD patient and whether any of the proposed pathophysiological mechanisms mentioned earlier are related to ***H. pylori*** infection. These particular questions were addressed in a recent study by Sarnelli and colleagues, in which they compared the symptom profile of NUD patients with and without ***H. pylori*** and looked at various pathophysiological mechanism including gastric emptying, sensation, and accommodation. They found no association between ***H. pylori*** infection and the overall prevalence of symptoms or the gastric sensorymotor functions.[10] This was confirmed in several other studies addressing each mechanism on its own, the discussion of which is beyond the scope of this review.[11,12]

## *H. PYLORI* TREATMENT IN NUD

The common rationale for H. pylori eradication in the absence of unequivocal evidence of the role of ***H. pylori*** in the pathogenesis of NUD, is on two grounds. Firstly, it is based on the presumption that we are missing a mechanism by which ***H. pylori*** causes NUD. Therefore, the rationale goes that if ***H. pylori*** eradication proves effective in improving symptoms, this in itself would be good enough evidence for justifying this approach. The other strategy is to prevent the occurrence of peptic ulcer disease that may develop in 10–15% of ***H. pylori***-positive patients, and more importantly, as mentioned earlier, prevent gastric cancer.

## *H. PYLORI* ERADICATION FOR NUD SYMPTOMS

The literature is rich when it comes to data related to this particular question and at least six to seven meta-analyses addressed this with different outcomes. The strongest evidence for the role of ***H. pylori*** eradication in symptomatic improvement in NUD patients comes from a Cochrane database review that was started in the year 2000 and continuously updated almost on a yearly basis, which consistently showed a small, but significant beneficial effect of ***H. pylori*** eradication reaching an average of 10% with a number needed to treat of 14 to cure or to markedly decrease symptoms in one patient. An economic model suggests that this modest benefit may still be cost-effective although further study is required.[13]

The problem with these meta-analyses is that the definition of NUD or its subtype was not clarified in a number of studies and the conclusions further compounded by the inclusion of patients with predominant reflux symptoms. The case was highlighted by the two meta-analyses published by Laine in 2001[14] and Gisbert ***et al.*** in 2002,[15] where the two authors found scant evidence to justify eradication of the organism to relieve dyspepsia. A stronger case against the eradication of ***H. pylori*** as an effective treatment for symptoms of NUD was further shown in analysis of two of the best randomized trials testing eradication of ***H. pylori*** in NUD and its effect on quality of life at the end of 1 year and showed no significant difference between the intervention and the placebo group. However, the secondary analysis revealed a small but significant benefit in patients with healed gastritis or with ulcer-type pain, rather than in patients with dysmotility-like pain groups.[16]

These meta-analyses mentioned above reveal the discordance between the results of various studies and underline the fact that there is no compelling evidence for the eradication of ***H. pylori*** in NUD patients. This is regardless of the cost effectiveness analysis of this approach, which is at best, of modest benefit in the most conservative analysis. This also is somewhat negated if we consider the resistance pattern that is known to occur more frequently in patients with NUD than when compared to patients with peptic ulcer disease.[17]

## PREVENTION OF GASTRIC CANCER

***H. pylori*** is considered as a class I carcinogen by WHO, as this was shown in different studies and meta-analyses to increase gastric cancer risk in H. pylori patients from 4 to 6 folds, and up to 20 folds in some populations.[18] The questions are: Does eradication of ***H. pylori*** prevent gastric cancer? And at what level is the intervention effective? These two questions were addressed by several studies and again the results were not consistent and no conclusive evidence that ***H. pylori*** eradication prevents gastric cancer was produced. Supportive data could, theoretically, be obtained from prospective trials of eradication, but, in practice, such trials do not exist and may be impossible to conduct for reasons of duration, ethics, etc. Although a recent meta-analysis aimed at studying the effect of ***H Pylori*** eradication in prevention of gastric cancer at the level of precancerous lesions and at an even earlier stage, the authors could only conclude that eradication of ***H. pylori*** is a plausible goal, but there is no evidence to support general screening programs for eradication.[19] Thus, it is difficult to justify treating 70% of the population harboring a low risk for gastric cancer for what appears to be a negligible true risk reduction that is not even proven on firm grounds.

## DISADVANTAGES OF ERADICATION OF *H. PYLORI*

In a population where the resistance for metronidazole is more than 78%[20] and the effectiveness of triple therapy is around 75%, there is a greater likelihood of subjecting patients to unwarranted side effects of treatment and increasing the risk of development of antibiotics resistance in the community. Furthermore, in case of treatment success, patients are put at a controversially increased risk of cardia cancer for which ***H. pylori*** colonization in the stomach is considered protective.[22]

In conclusion, despite the common occurrence of NUD and ***H. pylori*** in the community, justifying ***H. pylori*** eradication treatment based on the above-mentioned arguments is untenable. Additionally no definite benefit is achieved by this treatment either in terms of symptom improvement or in the prevention of gastric cancer.
